# Psychological effects of orthodontics treatment in adolescent and adults

**DOI:** 10.6026/9732063002001196

**Published:** 2024-09-30

**Authors:** Adel Alharbi

**Affiliations:** 1 Department of Orthodontic and Paediatric Dentistry, College of Dentistry, Qassim University, Saudi Arabia

**Keywords:** Dental aesthetics, orthodontic treatment, psychosocial impact, quality of life

## Abstract

Orthodontic treatment, primarily using fixed appliances, aims to correct malocclusion and improve dental function, appearance and psychological well-being. While the psychological effects of orthodontic treatment have been studied, there is limited research directly comparing these effects between adolescents and adults. Hence a cross-sectional design was used to compare the psychological impacts of orthodontic treatment between 100 adolescents (aged 13-17) and 100 adults (aged 26-35). Participants were recruited from orthodontic clinics, with data collection including demographic information and pre- and post-treatment psychological assessments using the Psychosocial Impact of Dental Aesthetics Questionnaire (PIDAQ). Statistical analyses involved unpaired t-tests and chi-square tests to compare outcomes and examine associations between categorical variables. Adolescents showed significant improvements post-treatment, with notable reductions in PIDAQ scores from 44.25 at baseline to 28.90 post-treatment. Similarly, reductions in dental self-confidence (DSC) scores, social impact (SI) scores and psychological impact (PI) scores were observed. Adults also demonstrated positive changes, with PIDAQ scores decreasing from 38.64 to 32.85, alongside reductions in DSC and SI scores. Although PI scores showed a slight decrease, overall improvements in dental aesthetics and psychosocial aspects were noted. Significant p-values (< 0.001) across all measured parameters highlighted age as a significant factor influencing treatment outcomes. Our study highlights the significant psychosocial benefits of orthodontic treatment across different age groups and cultural settings. Both adolescents and adults experience improvements in self-esteem, social interactions, and overall quality of life post-treatment.

## Background:

Malocclusions highly prevalent and affects an estimated 20-30% of the global population, making it a significant public health concern [1, 2]. Its multifactorial aetiology involves genetic, environmental, and functional factors [1]. Orthodontic treatment serves as the primary approach to correct malocclusion, improve dental function and appearance and potentially enhance psychological well-being and quality of life [3]. Current literature suggests that malocclusion can adversely impact self-esteem, social interactions, and overall quality of life, particularly during adolescence - a critical period marked by heightened self-consciousness and susceptibility to peer influence. During this stage, dental and facial aesthetics play a crucial role in self-perception and social acceptance [5, 5-6]. While the psychological effects of orthodontic treatment have been explored, there is a paucity of research directly comparing these effects between adolescents and adults undergoing such treatment. Adults seeking orthodontic treatment often have different motivations and life experiences, such as enhancing appearance for professional or personal reasons or boosting self-confidence after significant life events [5, 7]. The psychological impact of orthodontic treatment on adults may differ from that on adolescents due to varying levels of maturity, self-acceptance and life priorities [3]. This comparative study aims to investigate the psychological effects of orthodontic treatment on adolescent and adult populations by examining factors such as self-esteem, body image, social interactions and overall quality of life. Understanding the potential psychological benefits or challenges across different age groups can inform clinical practice, enabling healthcare professionals to provide better support and counsel patients throughout the treatment process.

## Materials & Methods:

## Study design: 

This cross-sectional study compared the psychological impacts of orthodontic treatment between 100 adolescents and 100 adults treated with fixed appliances. Participants were recruited from orthodontic clinics, with data collection including demographic information (age, gender) and pre- and post-treatment psychological assessments. The adolescent cohort comprised individuals aged 13-17; while the adult cohort included individuals aged 28-35. Exclusion criteria for both groups included prior orthodontic treatment, craniofacial anomalies and chronic medical or psychiatric conditions potentially affecting psychological well-being.

## Data collection:

To evaluate the psychological states of the patients, the Psychosocial Impact of Dental Aesthetics Questionnaire (PIDAQ) was utilized. The questionnaire specifically aims to assess the psychosocial impacts of orthodontic treatment, comprising 23 questions categorised into four subscales: social impact (SI) with 8 items, psychological impact (PI) with 6 items, aesthetic concern (AC) with 3 items and dental self-confidence (DSC) with 6 items. Responses were rated on a 5-point Likert scale from 0 (not at all) to 4 (very strong). Higher scores in the SI, PI and AC domains indicate greater psychological impact, while in the DSC domain, higher scores denote higher levels of self-confidence [9]. Baseline measurements were obtained before treatment initiation and post-treatment assessments were conducted after the completion of orthodontic procedures. Descriptive statistics (means, standard deviations) were calculated for age and psychological measures within each group.

## Ethical considerations:

The study protocol was reviewed and approved by an institutional review board or ethics committee. Informed consent was obtained from all participants (and parents/guardians of adolescents). Confidentiality and privacy of participant data were maintained throughout the study.

## Data analysis:

Statistical analysis involved unpaired t-tests to compare age and psychological outcomes between adolescents and adults. Additionally, chi-square tests were used to examine associations between categorical variables (gender, malocclusion type) and group membership. A significance level of p <0.05 was adopted to determine statistical significance. Data analysis was performed using SPSS 2.0 version.

## Results:

A total of 200 participants were enrolled in the study, comprising 100 adolescents (aged 13-17) and 100 adults (aged 26-35). The adolescent cohort, with an average age of approximately 15 years, exhibited significant improvements across multiple measures. It specifically, demonstrated a substantial decrease in the PIDAQ scores, from 44.25 at baseline to 28.90 post-treatment. With notable reductions in DSC scores (from 16.63 to 10.11), SI scores (from 14.05 to 9.12), and PI scores (from 13.60 to 9.69) (Table 1, Table 2 and Table 3). The adult cohort, averaging around 30 years old, also demonstrated positive changes following treatment. Adults experienced a decrease in PIDAQ scores (from 38.64 to 32.85), reductions in DSC scores (from 14.60 to 10.75) and SI scores (from 12.19 to 10.74). Although there was a slight decrease in PI scores (from 11.85 to 11.36), overall improvements in dental aesthetics and psychosocial aspects were noted post-treatment (Table 4, Table 5 and Table 6). Statistical analyses confirmed these differences, with significant p-values (<0.001) across all measured parameters, highlighting age as a significant factor influencing treatment outcomes in orthodontics (Table 7). Additionally, crosstab analyses revealed no significant differences in the distribution of malocclusion types or gender between adolescents and adults (Table 8 and Table 9).

## Discussion:

Our research examined the effect of orthodontic care on the psychological and social health of 200 individuals, including adolescents aged 13-17 and adults aged 26-35. The results demonstrated significant enhancements in psychological well-being post-treatment. Adolescents in our study, averaging around 15 years of age, demonstrated significant reductions in the PIDAQ scores following orthodontic treatment. This aligns with previous research indicating that orthodontic intervention positively influences adolescents' self-esteem and social confidence [8, 9]. Previous studies have consistently shown that younger patients often experience notable improvements in self-perception and social interactions because of enhanced dental aesthetics after treatment. Additionally, adolescents tend to prioritise aesthetic outcomes and derive substantial psychological benefits from undergoing orthodontic care [9]. In contrast, the adult cohort in our study, averaging approximately 30 years old, also showed improvements in PIDAQ scores post-treatment. Studies in the literature support our findings by underscoring the positive impact of orthodontic treatment on adult patients' quality of life and psychological well-being [10, 11]. Research indicates a substantial increase in self-esteem and social confidence among adults undergoing orthodontic care, highlighting improvements in emotional well-being and social interactions [10]. Additionally, studies have reported significant enhancements in psychological attributes and overall quality of life after orthodontic treatment [11].

Furthermore, our study delved into cultural influences specific to the Indian population, including assessments of functional limitations and matrimonial concerns related to dental aesthetics. This approach builds upon existing research highlighting how cultural factors influence patient motivations and treatment outcomes [12, 13]. Cultural norms and societal expectations significantly shape patients' perceptions of dental aesthetics and treatment priorities, particularly in terms of social interactions and self-confidence [12]. Previous studies have similarly emphasised the critical role of cultural context in shaping patients' psychosocial experiences and treatment outcomes, underscoring the necessity for culturally sensitive approaches in orthodontic care [13]. This study's strengths include its comparative design and the use of the validated PIDAQ to ensure a reliable and comprehensive assessment of psychosocial factors. However, limitations include the cross-sectional design and the reliance on self-reported measures. In summary, our findings underline the significant psychosocial benefits of orthodontic treatment across different age groups and cultural settings. By integrating findings from previous studies, our research contributes to the broader understanding of how orthodontic care enhances quality of life and social well-being, supporting its role in improving patients' psychosocial outcomes globally. Future research should continue to explore these outcomes in diverse populations to optimise orthodontic interventions and enhance patient satisfaction.

## Conclusion:

This study investigated the comparative psychosocial impact of orthodontic treatment on adolescents and adults using the PIDAQ. The findings revealed significant improvements in psychosocial well-being, as evidenced by lower PIDAQ scores, in both age groups after undergoing orthodontic treatment. However, the adolescent cohort experienced a more profound positive impact, with greater reductions in PIDAQ scores compared to the adult cohort. These results highlight the vulnerability of adolescents to the psychosocial consequences of malocclusion and the potential benefits of addressing dental aesthetics concerns during this critical developmental stage. The study further elucidated the psychosocial impact of orthodontic treatment, demonstrating that individuals with more severe malocclusion experienced greater improvements in PIDAQ scores, irrespective of age. This underscores the importance of timely intervention to mitigate the negative psychosocial effects of significant dental irregularities. While no gender differences were observed within each cohort, the multifaceted nature of the psychosocial benefits, encompassing dental self-confidence, social impact and psychological well-being, was evident across both age groups. These findings have important clinical implications, emphasising the need for orthodontists and healthcare professionals to acknowledge and address the psychosocial aspects of malocclusion, particularly in adolescent patients, to enhance overall treatment outcomes and well-being.

## Figures and Tables

**Table 1:** Descriptive statistics for adolescent group

	**N**	**Minimum**	**Maximum**	**Mean**	**Std. Deviation**
Age	100	13	17	14.98	1.40691
Baseline PIDAQ	100	37	52	44.25	4.03363
Post-Tx PIDAQ	100	24	34	28.9	2.60729
Baseline DSC	100	13	20	16.63	1.5677
Post-Tx DSC	100	8	13	10.11	1.24637
Baseline SI	99	12	16	14.051	1.16386
Post-Tx SI	99	7	10	9.1212	0.90658
Baseline PI	99	12	16	13.596	1.4563
Post-Tx PI	99	9	11	9.6869	0.6332
DSC: Dental Self-Confidence, PI: Psychological Impact,
PIDAQ: Psychosocial Impact of Dental Aesthetics Questionnaire, SI: Social Impact

**Table 2:** Frequency distribution of gender in adolescent group

**Gender**	**Frequency**	**Percent**	**Valid Percent**	**Cumulative Percent**
Male	50	50	50	50
Female	50	50	50	100
Total	100	100	100	

**Table 3:** Frequency distribution of malocclusion types in adolescent

**Malocclusion**	**Frequency**	**Percent**	**Valid Percent**	**Cumulative Percent**
Class I	41	41	41	41
Class II	39	39	39	80
Class III	20	20	20	100
Total	100	100	100	

**Table 4:** Descriptive statistics for adult group

	**N**	**Minimum**	**Maximum**	**Mean**	**Standard Deviation**
Age	100	26	35	30.02	2.76698
Baseline PIDAQ	100	35	42	38.64	1.74957
Post-Tx PIDAQ	100	30	36	32.85	1.5333
Baseline DSC	100	13	16	14.6	0.964
Post-Tx DSC	100	10	12	10.75	0.78335
Baseline SI	100	11	14	12.19	0.70632
Post-Tx SI	100	9	12	10.74	0.52455
Baseline PI	100	11	12	11.85	0.35887
Post-Tx PI	100	11	12	11.36	0.48242
DSC: Dental Self-Confidence, PI: Psychological Impact,
PIDAQ: Psychosocial Impact of Dental Aesthetics Questionnaire, SI: Social Impact

**Table 5:** Frequency distribution of gender in adult groups

**Gender**	**Frequency**	**Percent**	**Valid Percent**	**Cumulative Percent**
Male	51	51	51	51
Female	49	49	49	100
Total	100	100	100	

**Table 6:** Frequency distribution of malocclusion types in adult groups

**Malocclusion**	**Frequency**	**Percent**	**Valid Percent**	**Cumulative Percent**
Class I	35	35	35	35
Class II	33	33	33	68
Class III	32	32	32	100
Total	100	100	100	

**Table 7:** Comparative analysis of baseline and post-treatment measures between adolescent and adult groups

	**Group**	**N**	**Mean**	**Std. Deviation**	**P value**
Age	Adolescent	100	14.98	1.40691	0
	Adult	100	30.02	2.76698	
Baseline PIDAQ	Adolescent	100	44.25	4.03363	0
	Adult	100	38.64	1.74957	
Post-Tx PIDAQ	Adolescent	100	28.9	2.60729	0
	Adult	100	32.85	1.5333	
Baseline DSC	Adolescent	100	16.63	1.5677	0
	Adult	100	14.6	0.964	
Post-Tx DSC	Adolescent	100	10.11	1.24637	0.001
	Adult	100	10.75	0.78335	
Baseline SI	Adolescent	99	14.051	1.16386	0
	Adult	100	12.19	0.70632	
Post-Tx SI	Adolescent	99	9.1212	0.90658	0
	Adult	100	10.74	0.52455	
Baseline PI	Adolescent	99	13.596	1.4563	0
	Adult	100	11.85	0.35887	
Post-Tx PI	Adolescent	99	9.6869	0.6332	0.005
	Adult	100	11.36	0.48242	
DSC: Dental Self-Confidence, PI: Psychological Impact,
PIDAQ: Psychosocial Impact of Dental Aesthetics Questionnaire, SI: Social Impact

**Table 8:** Crosstab analysis of malocclusion types across adolescent and adult groups

			**Group Adolescent**	**Adult**	**Total**	**P value**
Malocclusion	Class I	Count	41	35	76	
		% within Malocclusion	53.90%	46.10%	100.00%	
	Class II	Count	39	33	72	
		% within Malocclusion	54.20%	45.80%	100.00%	
	Class III	Count	20	32	52	0.154
		% within Malocclusion	38.50%	61.50%	100.00%	
Total		Count	100	100	200	
		% within Malocclusion	50.00%	50.00%	100.00%	

**Table 9:** Crosstab analysis of gender across adolescent and adult groups

			**Group Adolescent**	**Adult**	**Total**	**P value**
Gender	Male	Count	50	51	101	
		% within Gender	49.50%	50.50%	100.00%	
	Female	Count	50	49	99	0.5
		% within Gender	50.50%	49.50%	100.00%	
Total		Count	100	100	200	
		% within Gender	50.00%	50.00%	100.00%	
